# mRNA modification orchestrates cancer stem cell fate decisions

**DOI:** 10.1186/s12943-020-01166-w

**Published:** 2020-02-26

**Authors:** Weicheng Liang, Zexiao Lin, Cong Du, Dongbo Qiu, Qi Zhang

**Affiliations:** 1 Vaccine Research Institute, The Third Affiliated Hospital of Sun Yat-sen University, Sun Yat-sen University, Guangzhou, China; 2 Guangdong Provincial Key Laboratory of Liver Disease Research, Guangzhou, China; 3 Cell-gene Therapy Translational Medicine Research Center, The Third Affiliated Hospital of Sun Yat-sen University, Guangzhou, China; 4 Department of Medical Oncology, The Third Affiliated Hospital of Sun Yat-sen University, Sun Yat-sen University, Guangzhou, China

**Keywords:** RNA modification, Cancer stem cells, 5-methylcytosine, N^6^-methyladenosine, A-to-I editing

## Abstract

Despite their small numbers, cancer stem cells play a central role in driving cancer cell growth, chemotherapeutic resistance, and distal metastasis. Previous studies mainly focused on how DNA or histone modification determines cell fate in cancer. However, it is still largely unknown how RNA modifications orchestrate cancer cell fate decisions. More than 170 distinct RNA modifications have been identified in the RNA world, while only a few RNA base modifications have been found in mRNA. Growing evidence indicates that three mRNA modifications, inosine, 5-methylcytosine, and N^6^-methyladenosine, are essential for the regulation of spatiotemporal gene expression during cancer stem cell fate transition. Furthermore, transcriptome-wide mapping has found that the aberrant deposition of mRNA modification, which can disrupt the gene regulatory network and lead to uncontrollable cancer cell growth, is widespread across different cancers. In this review, we try to summarize the recent advances of these three mRNA modifications in maintaining the stemness of cancer stem cells and discuss the underlying molecular mechanisms, which will shed light on the development of novel therapeutic approaches for eradicating cancer stem cells.

## Introduction

With the rapid development of high-throughput sequencing technologies, more than 170 types of post-transcriptional RNA modifications have been detected so far [1]. RNA modifications were first identified in non-coding RNA elements like tRNA and rRNA [2] and have been historically regarded as irreversible decorations on RNA bases. However, subsequent investigations showed that some RNA modifications are actually reversible [3, 4]. Moreover, emerging evidence demonstrates that these dynamic and reversible RNA modifications are widely present in various RNA molecules, not only non-coding RNA but also mRNA. The multitude of RNA modifications led to the birth of “RNA epigenetics” in 2010 [5] and the “Epitranscriptome” in 2012 [6], which are analogous to the concept of epigenetic modulation mediated by DNA or histone modifications.

Emerging RNA immunoprecipitation-sequencing methods have provided a detailed understanding of the genome-wide landscape of RNA modifications in human cells [7–12]. However, the majority of these modifications are mapped to tRNA and rRNA [13]. So far, only a few forms of RNA modifications have been identified in mRNA, such as N^6^-methyladenosine (m^6^A), N^1^-methyladenosine(m^1^A), Inosine (I), Pseudouridine (Ψ), 5-methylcytosine (m^5^C), 5-hydroxymethylcytidine (hm^5^C), N^6^,2′-O-dimethyladenosine (m^6^Am), 7-methylguanosine (m^7^G), and N^4^-acetylcytidine (ac^4^C). Despite the low frequency in the human genome, they affect almost every step of mRNA biogenesis and degradation. For example, mRNA modifications extensively modulate a vast pool of biochemical events surrounding mRNA metabolisms, such as mRNA splicing [8, 14], RNA folding [15, 16], stability [17–21], mRNA translation [22–24], and RNA transport [25, 26].

Growing evidence indicates that mRNA modifications display dramatic and dynamic variations during lineage commitment and cell reprogramming [27–29], suggesting their biological significance in the maintenance of cell identity. As oncogenic transformation frequently accompanies activation of pluripotency genes like NANOG, MYC, and Oct4 [30–32], it is likely that mRNA modifications also actively participate in modulating cancer cells’ fate through controlling these oncogenic factors. Consistently, subsequent studies have found that mRNA modifications are also essential for maintaining the stemness and malignancy of cancer stem cells [33–36].

The concept of cancer stem cells (CSCs) was proposed in the 1970s [37]. Analogous to stem cells in healthy tissue, CSCs possess stem-like properties, including the capacity for self-renewal and the ability to enhanced tumor initiation upon experimental transplantation [38]. It is proposed that the existence of this small but aggressive cell population possesses a high risk of drug resistance and tumor relapse [39, 40]. The CSC hypothesis posits that tumors mirror the hierarchy as normal tissues and that the CSCs are located at the apex of this hierarchical organization [41, 42]. With the elevated capacity of persistent proliferation, CSCs undergo asymmetric division, leading to complicated tumor heterogeneity and resistance to chemotherapy [43].

A recent breakthrough of the high-throughput sequencing platform has illustrated the detailed epigenetic landscape in CSCs [44]. The epigenetic modifications of DNA or histones are fundamental to the maintenance of cancer stem cell identity [45–48]. For instance, transformed cells which escape the senescence checkpoint, possess elevated levels of DNA methylation, leading to enhanced self-renewal and pro-survival signals [45].

However, as a novel modification form in the field of epigenetics, the function of mRNA modification in controlling the stemness of CSCs is still poorly understood. Currently, the three widespread mRNA modification forms are inosine, 5-methylcytosine (m^5^C), and N^6^-methyladenosine (m^6^A). In this review, we will provide an update of how these three mRNA modifications orchestrate regulatory gene networks within CSCs (Table 1). In addition, we will discuss their underlying molecular mechanisms and potential novel therapeutic strategies based on mRNA modification profiles.

**Table 1:** A summary of mRNA modification and cancer stem cells

Cancer cell types	RNA modification	Expression profiles in CSC	Molecular mechanisms	References
Leukemia	A-to-I	Increased	A-to-I editing induced alternative splicing of GSK3β, resulting in enhanced β-catenin expression	[49, 50]
Multiple myeloma	A-to-I	Increased	A-to-I editing occurred in the exon of GLI1 mRNA, leading to a novel GLI1 protein with a point mutation	[51]
Leukemia	A-to-I	Increased	A-to-I editing occurred in the 3’UTR of MDM2 mRNA and miR-155 would no longer bind to the edited 3’UTR region	[52]
Leukemia	A-to-I	Increased	A-to-I editing in let-7 precursor impaired let-7 biogenesis	[36]
Skin cancer	m^5^C	Decreased	NSUN2-deletion impaired protein synthesis	[53]
Breast cancer	m^6^A	Decreased	ALKBH5 reduced m^6^A level of NANOG, which stabilized NANOG mRNA	[33]
Glioblastoma	m^6^A	Decreased	Knockdown of METTL3 or METTL14 in CSCs increased the expression of ADAM19 and EPHA3	[34]
Glioblastoma	m^6^A	Decreased	ALKBH5 demethylated FOXM1 mRNA transcripts and stabilized FOXM1	[35]
Glioblastoma	m^6^A	Increased	SOX2 was a target for METTL3 and methylated SOX2 mRNA displayed prolonged stability	[54]
Leukemia	m^6^A	Decreased	Treatment with FTO inhibitor R-2HG induced the degradation of MYC/CEBPA mRNAs	[55]
Leukemia	m^6^A	Increased	METTL14 catalyzed the m^6^A modification in oncogenic factors MYC and MYB, increasing their mRNA stability	[56]

## A-to-I modification and cancer stem cells

In eukaryotes, adenosine-to-inosine (A-to-I) editing is one of the most prevalent RNA modifications. This process involves hydrolytic deamination of adenosine, catalyzed by the ADAR family members (ADAR1, ADAR2, and ADAR3) [57]. The newly generated inosine base is interpreted by the ribosome as guanosine during mRNA translation, leading to altered protein products, if the modification occurs in the protein-coding region [58]. Among the ADAR family members, ADAR1 and ADAR2 are ubiquitously expressed in eukaryotic cells while ADAR3 is highly expressed in brain cells [59]. Genetic ablation of ADAR1 in mice led to embryonic lethality, at embryonic day E12.5, due to severe global interferon response and defects in hematopoiesis [60, 61]. ADAR2-deficient mice were born at the normal Mendelian ratio and appeared to develop normally, but these mice died within 3 weeks after birth, during or soon after weaning [62]. These results suggest that ADAR1 and ADAR2 are indispensable for embryonic development and normal growth. According to a large-scale study including 6236 patient samples from 17 cancer types, A-to-I modifications display distinct distribution patterns in tumors and normal tissues [63]. However, most modifications existed in the non-coding regions of the mRNA [63]. Despite their prevalence, the functional consequences of these aberrant patterns in tumorigenesis remain elusive.

**Fig. 1:**
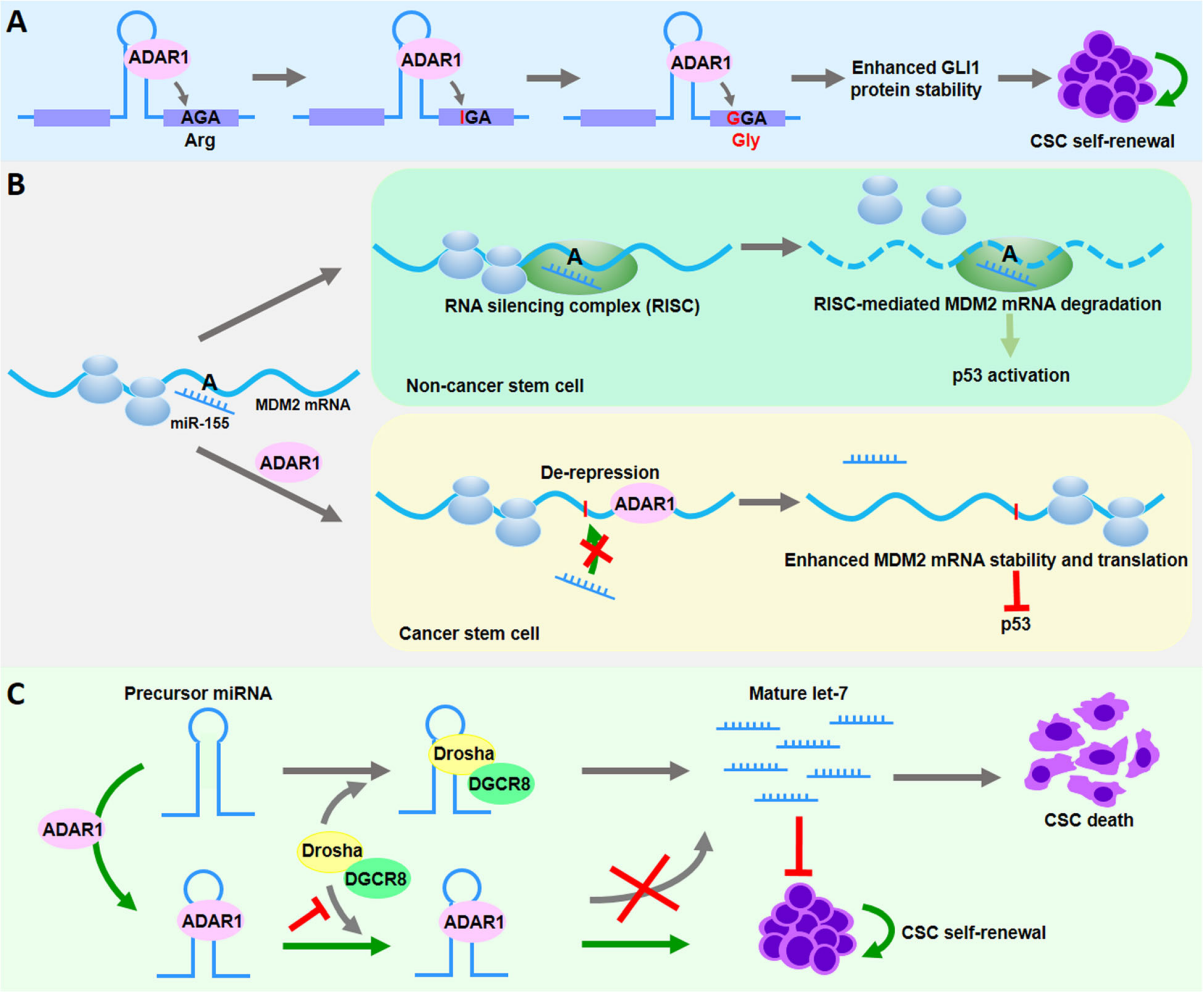
Functional implication of ADAR1-mediated A-to-I editing in cancer stem cells. **a** A-to-I editing in the exon 12 of GLI1 transcript results in coding sequence change from Arg to Gly at position 701, which stabilizes GLI1 protein and enhances cancer stem cell renewal. **b** A-to-I editing at the 3′ UTR of MDM2 alters the interaction between MDM2 mRNA transcript and miR-155. **c** A-to-I RNA editing impairs the miRNA biogenesis of tumor suppressor let-7 through altering pre-miRNA secondary structures, leading to escape from let-7-mediated cancer cell death

Previous studies demonstrated that A-to-I modifications could modulate the stemness of hematopoietic malignancies. For example, ectopic expression of ADAR1 potentiated malignant myeloid progenitor expansion through promoting alternative splicing of GSK3β, which enhanced the production of a misspliced form of GSK3β [49]. In vivo studies showed that leukemia progenitor cells harboring this misspliced GSK3β gene displayed enhanced β-catenin expression, which was required for the self-renewal of leukemia stem cells (LSCs) [50]. It was estimated that the genomic amplification of ADAR1 occurred in 30–50% of multiple myeloma patients and portended an unfavorable prognosis [51]. The silencing of ADAR1 attenuated in vivo engraftment of myeloma through suppression of the transcriptional activity of GLI1 [51], a self-renewal agonist and candidate marker for CSCs [64]. Further studies have shown that ADAR1 can edit exon 12 of the GLI1 transcript, leading to a novel GLI1 protein with a point mutation (Fig. 1a), which might stabilize the GLI1 protein by preventing the binding of a Hedgehog pathway negative regulator [51]. When comparing the A-to-I editing status, scientists revealed the elevated frequency of 3’UTR editing events in malignant progenitor cells. Interestingly, the majority of A-to-I events occurred in the 3’UTR of MDM2 RNA transcripts [52]. As an E3 ubiquitin ligase, MDM2 directly associates with and subsequently inactivates the transactivation domain of tumor suppressor p53. When the A-to-I editing occurred in the 3’UTR of MDM2 transcripts, miR-155 no longer bound to the edited 3’UTR (Fig. 1b), leading to the stabilization of MDM2 and inactivation of p53 [52].

In addition to mRNA, growing evidence demonstrates that ADAR1 also hinders the biogenesis of tumor-suppressive miRNAs, thereby driving leukemia stem cell self-renewal. Wild-type ADAR1, but not the editing-defective ADAR1^E912^ mutant, potentiates self-renewal gene expression and suppresses the biogenesis of stem cell inhibitory microRNA let-7 [36]. Subsequent studies found that A-to-G nucleotide changes altered RNA secondary structures at the Drosha/DGCR8 cleavage sites, leading to impaired let-7 miRNA biogenesis (Fig. 1c).

Recent studies have provided substantial new insights into how A-to-I modifications regulate RNA splicing. mRNA maturation involves serial processing steps which structurally alter the newly synthesized RNA transcripts, such as 5′ end capping, RNA splicing, RNA editing, and 3′ end polyadenylation. Among these molecular processes, RNA splicing is a well-documented molecular event that is tightly regulated by A-to-I modification [65, 66]. The creation or removal of splice sites by A-to-I editing plays a vital role in the RNA splicing process. In mammalian cells, A-to-I modifications preferentially occur at Alu elements in the introns of the transcribed gene and create novel splice sites, resulting in exonization of the noncoding sequence. According to high-throughput sequencing results, it was estimated that around 1.4% of total human mRNAs are subject to A-to-I editing and that the editing sites are closely associated with RNA splicing machinery [67]. Another mechanism by which A-to-I editing affects splicing is mediated through altering RNA secondary structures [68]. Because both ADAR proteins and splicing machinery act on double-stranded RNA, the substitution of adenosine by inosine may change the stability of the RNA duplex [69], and eventually alter the mutual interaction between the splicing machinery and the double-stranded RNAs.

In addition, emerging evidence suggests that A-to-I editing plays a role in regulating RNA stability. In human B cells, DNA and RNA sequencing data showed that the expression levels of thousands of genes were modulated by ADAR proteins [70]. Furthermore, ADAR1 strengthened target RNA stability through physically interacting with HuR, a potent RNA stabilizer. In mouse cells, a similar finding was also reported for ADAR2. The unedited Ctn RNA displayed a higher binding affinity with the RNA destabilizers HuR and PARN when compared to the ADAR2-edited Ctn RNA [71]. However, ADAR2-mediated A-to-I editing of the 3’UTR of Ctn RNA hampered the interaction between the RNA destabilizer and Ctn RNA transcript, thereby leading to a prolonged half-life of Ctn RNA. Although the current findings indicate that both ADAR1 and ADAR2 promote RNA stability through HuR, HuR can function both as an RNA destabilizer or an RNA stabilizer, the mechanism of which is still largely unknown.

In summary, ADAR1 plays a pivotal role in maintaining the stemness of hematopoietic malignancies. Through enhancing self-renewal gene expression and impairing the biogenesis of tumor-suppressive miRNAs, ADAR1 is indispensable for normal hematopoietic stem cell maintenance and leukemia stem cell self-renewal. This suggests that ADAR1 may play an important role in a wide spectrum of hematopoietic disorders which have acquired aberrant stem cell self-renewal features.

## m^5^C modification regulates cancer stem cells

**Fig. 2:**
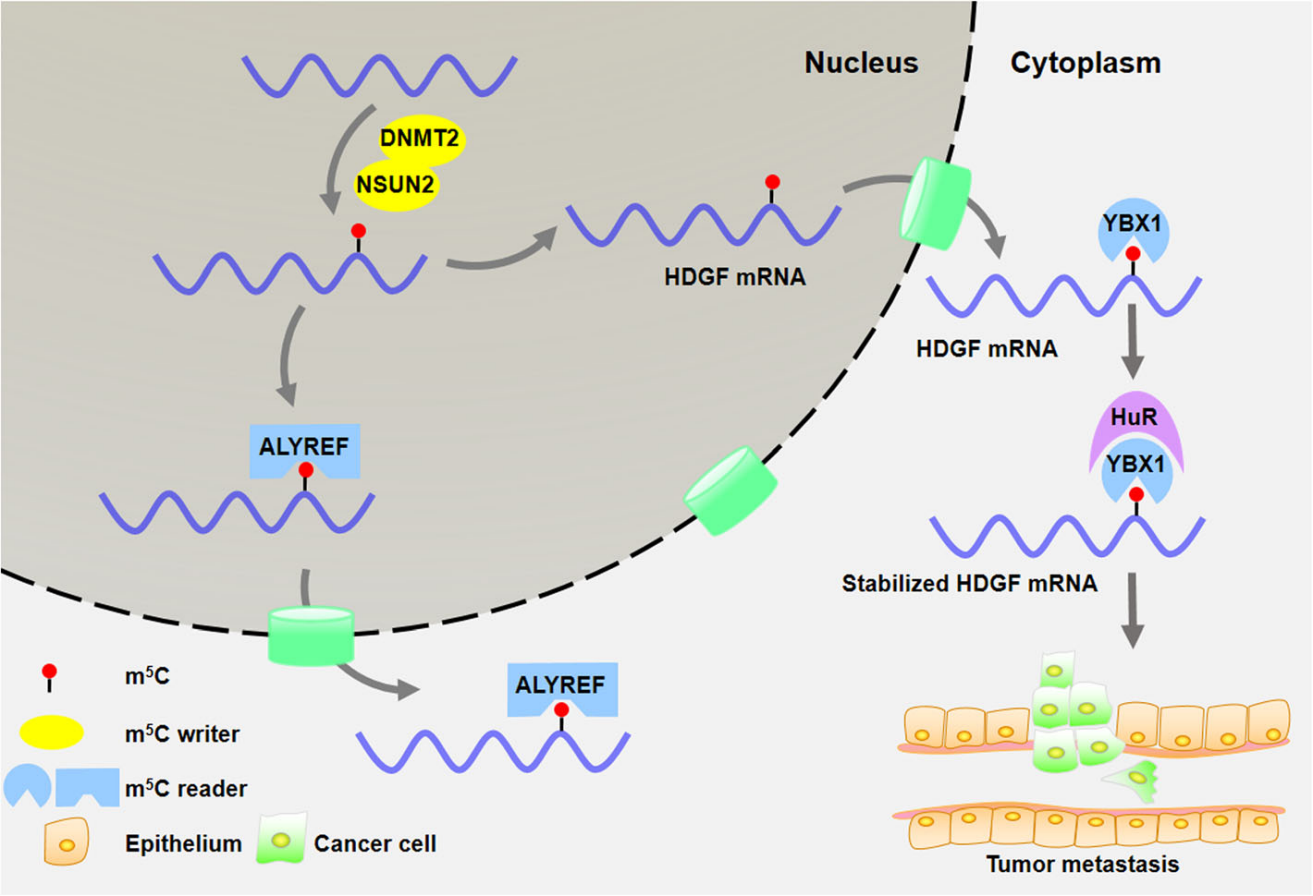
Roles of m^5^C RNA modifications in cancer. DNMT2 and NSUN2 are RNA methyltransferases responsible for m^5^C modification. The m^5^C reader protein ALYREF recognizes m^5^C- methylated mRNA and initiates transportation from nucleus towards cytoplasm. In bladder cancer, HDGF mRNA is methylated and captured by reader protein YBX1. By interacting with YBX1, HuR stabilizes HDGF mRNA and induces tumor metastasis

5-methylcytosine (m^5^C) was first identified in tRNA and rRNA [72, 73]. Recently, the transcriptome-wide landscape of the m^5^C profile has shown that m^5^C modifications are preferentially located in the vicinity of the translational start codon of mRNA [74]. In addition, the m^5^C modification is predominantly catalyzed by the RNA methyltransferase, NSUN2, and the m^5^C sites are recognized by the m^5^C reader protein, ALYREF (Fig. 2) [75]. Besides mRNA, NSUN2 also catalyzes tRNA methylation at the variable loop region (C47-C50) [76]. In NSUN2-null mice, the m^5^C modification was lost in tRNA at the following positions, tRNA^Gly^, tRNA^Leu^, tRNA^Asp^, and tRNA^Val^ [77]. In addition to NSUN2, DNMT2 is another confirmed m^5^C RNA methyltransferase [78, 79], which mainly catalyzes tRNA methylation [77, 80].

NSUN2 is highly expressed in various types of solid tumors [81–83] and is transcriptionally activated by the MYC oncogene [84]. In bladder cancer, many oncogenic RNAs harbor hyper-methylated m^5^C sites, which are catalyzed by NSUN2 [85]. YBX1 recognizes m^5^C-modified mRNA and then recruits mRNA stability maintainer, HuR (Fig. 2), which subsequently stabilizes the putative oncogene HDGF in an m^5^C-dependent manner [85].

However, paradoxically, in skin cancer, NSUN2 expression was downregulated and the depletion of NSUN2 increased the population of tumor-initiating cells [53]. By quantifying protein synthesis, it was found that tumor-initiating cells synthesized less protein when compared to their progeny [53]. Thus, a reduction in protein translation is beneficial for the generation of tumor-initiating cells as well as other stem cell types [53, 86].

The above contradictory findings raised two fundamental questions for NSUN2 and m^5^C methylation in cancer biology: (1) does NSUN2 exert opposite roles in different cancer types, and (2) does m^5^C methylation either promote or inhibit protein translation based on different microenvironments? It is not rare that a single gene can have dual roles as either a tumor suppressor or an oncogene. For instance, the stem cell marker gene, KLF4, has contrasting roles in various cancer types as reported in previous studies [87, 88]. Because different types of cancer possess distinct contexts and NSUN2 can target multiple RNAs simultaneously, it is likely that NSUN2 exerts its pleiotropic roles in a context-dependent pattern. In other words, if NSUN2 predominantly targets oncogenic RNA molecules in specific types of cancer, it would function as an oncogene. Otherwise, it would act as a tumor suppressor if it mainly affects tumor-suppressive RNA molecules.

Besides, whether the m^5^C modification promotes or suppresses mRNA translation is still under debate. It was reported that NSUN2-mediated m^5^C modification can either promote CDK1 and IL-17A translation or attenuate p27^KIP1^ translation [89–91]. In the DNMT2/NSUN2 double knockout cells, overall protein synthesis was dramatically reduced whereas protein translation in single knockout cells was not affected [77].

Taken together, these contrasting findings indicate that m^5^C has a sophisticated role in governing mRNA translation and further investigation is necessary to further clarify its mechanism of action.

## m^5^C and the cellular fate of mRNA

Currently, it remains largely unknown how m^5^C modification alters mRNA expression. Recent findings suggest that m^5^C might enhance mRNA stability. It was found that YBX1 preferentially recognized mRNA with m^5^C modifications and subsequently stabilized target mRNAs. In zebrafish early embryos, m^5^C-modified maternal mRNAs displayed enhanced stability when compared to non-m^5^C-modified mRNAs [92]. Subsequent mechanistic studies showed that YBX1 enhanced the stability of m^5^C-modified mRNAs through cooperation with mRNA stabilizer Pabpc1a. This highlights an essential role of m^5^C modification in RNA metabolism and zebrafish embryo development. In human bladder cancer cells, it was reported that many oncogenic mRNAs were hypermethylated by NSUN2. As an m^5^C reader, YBX1 recognized m^5^C sites within HDGF mRNA transcripts and then recruited mRNA stabilizer HuR, leading to enhanced mRNA stability [85]. High expression of oncogenic HDGF mRNA subsequently promoted the pathogenesis of bladder cancer.

Besides, some recent papers indicate that m^5^C affects not only mRNA stability but also mRNA splicing. It was reported that the distribution of m^5^C sites partially overlapped with the binding sites of some RNA binding proteins. By analyzing the PAR-CLIP data from public databases, scientists uncovered that m^5^C sites were enriched in the binding regions of the mRNA splicing factors SRSF3 and SRSF4 [93], indicating a potent role of m^5^C modification in modulating mRNA alternative splicing. Interestingly, a recent publication revealed a previously unknown role of m^5^C modification in HIV infection through modulation of RNA splicing and translation [94]. It was found that HIV-1 RNA transcripts were highly methylated by m^5^C methyltransferase NSUN2. High-throughput sequencing data subsequently confirmed an m^5^C site located in the vicinity of the A2 splice site within the Vif gene. Knockout of NSUN2 reduced the use of the D1/A2 splice junction and altered the RNA splicing of HIV RNA transcript. Moreover, loss of NSUN2 reduced m^5^C occurrence on HIV RNA transcripts and hampered HIV RNA translation, suggesting an important role of m^5^C modification in the life cycle of HIV. Collectively, these data suggest that m^5^C modification may be involved in mRNA splicing, although there are many questions that must be addressed by scientists in the coming future. For instance, it is still unclear which reader proteins recognize m^5^C sites and thereby mediate mRNA splicing. Since only a small number of mRNA splicing factor binding sites overlap with the m^5^C region, is it a specific phenomenon that occurs in some particular RNA transcripts? Further studies are needed to elucidate the functional role of m^5^C in alternative splicing.

High-throughput sequencing has provided a detailed mapping of m^5^C sites in eukaryotic cells. Moreover, the identification of m^5^C writers and readers has aided our understanding of the functional roles of m^5^C modifications in the regulation of RNA stability, alternative splicing, and RNA translation. Although scientists have illustrated the genome-wide m^5^C distribution at single-nucleotide resolution, the role of m^5^C in mammalian cells remains unclear. For instance, the m^5^C eraser is still unknown and how m^5^C mediates other RNA processing steps is to be further explored. Therefore, it is necessary to identify novel m^5^C-interacting proteins or enzymes, which will further elucidate the functional roles of m^5^C in various biological events and human diseases.

## The dual role of m^6^A in cancer stem cell

N^6^-methyladenosine (m^6^A), occurring at the N^6^ position of adenosine, is the most pervasive and abundant post-transcriptional modification in eukaryotic cells. By using the antibody-enrichment sequencing method, m^6^A sites were found in all areas of mRNA transcripts but displayed significant enrichment near the stop codon and 3’UTR region [95]. It was estimated that mRNA transcripts from 7676 mammalian genes have m^6^A modification [95]. Subsequent studies revealed that 77.29% of m^6^A sites are present in a consensus motif DRACH (D = A, G or U; R = A or G; H = A, C or U) [96]. Further bioinformatics analysis demonstrates that m^6^A RNA modification is evolutionarily conserved across different species [97].

**Fig. 3:**
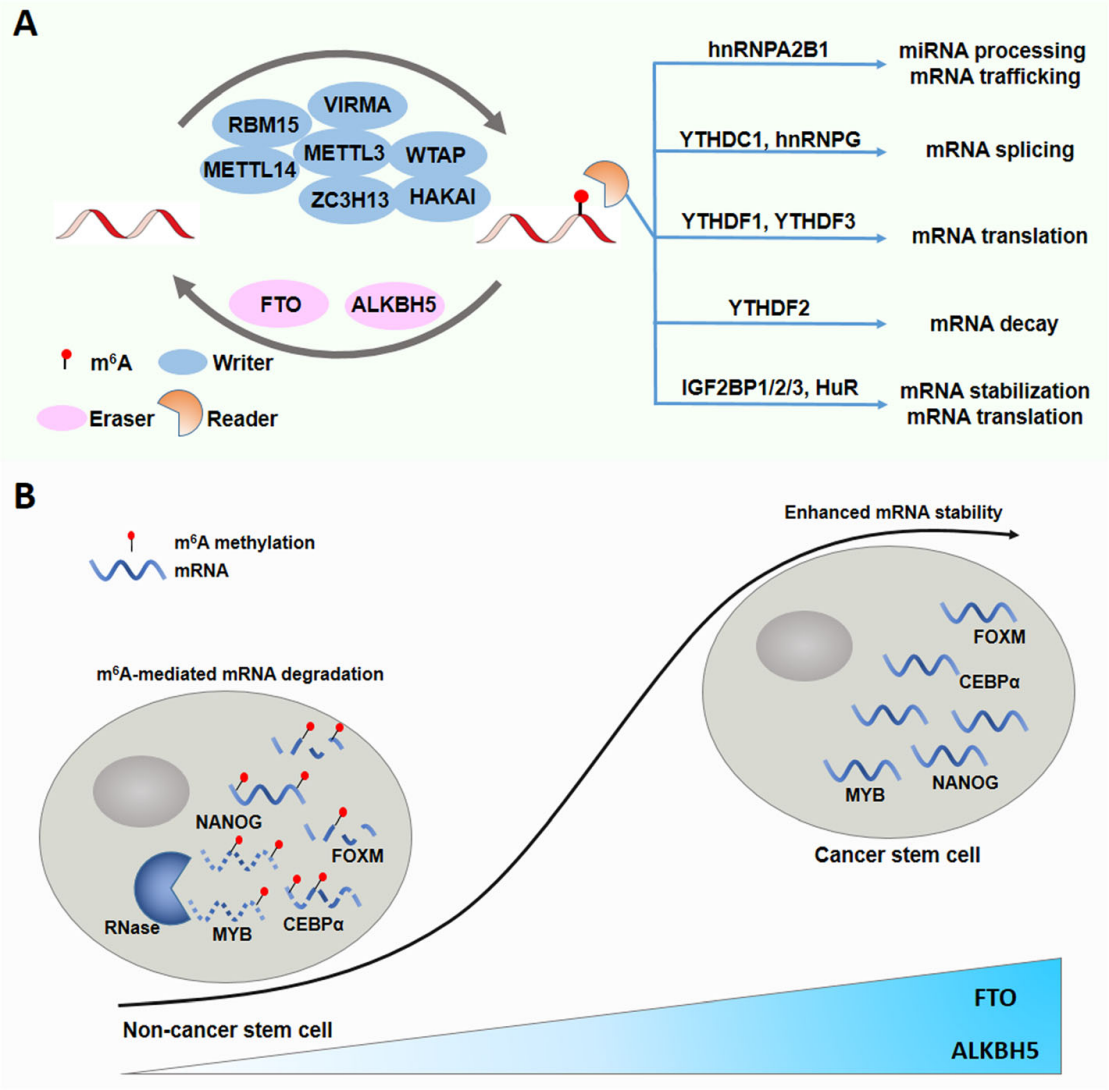
The functional role of m^6^A modification in cancer stem cells. **a** Summary of m^6^A modification machinery. The m^6^A effectors include the writer proteins (METTL3/METTL14/WTAP complex, probably also of VIRMA and RBM15, etc.), eraser proteins (m^6^A RNA demethylases: FTO and ALKBH5), and reader proteins (YTHDC1, YTHDF1/2/3, hnRNPA2B1, hnRNPG, IGF2BP1/2/3, HuR). **b** m^6^A affects mRNA stability and cancer stem cell differentiation. In cancer stem cells, FTO and ALKBH5 are highly expressed and remove m^6^A methylation on cancer stem cell marker genes like NANOG and MYB, leading to the stabilization of target mRNAs and enhanced self-renewal capacity

The deposition of m^6^A in mRNA is mediated by methyltransferase complexes, such as METTL3/14, VIRMA, RBM15/15B, WTAP, HAKAI, and ZC3H13, called ‘writers’. The removal of m^6^A from mRNA transcript is catalyzed by ‘eraser’ demethylases, FTO and ALKBH5 (Fig. 3a). Owing to the presence of writers and erasers, m^6^A modification is a dynamic and reversible process that can fine-tune the fate of mRNA transcripts within a short time. This important characteristic allows prompt adaptation to abrupt environmental changes, such as hypoxia and injury.

In the cytoplasm, m^6^A sites are recognized by m^6^A binding proteins, such as hnRNPA2B1, YTHDF1/2/3, YTHDC2, and IGF2BP1/2/3, called ‘readers’. Recent advances highlight m^6^A readers as fundamental players in the modulation of mRNA metabolism (Fig. 3a). It has been reported that the binding of hnRNPA2B1 to m^6^A sites promotes primary miRNA processing and mediates the nucleocytoplasmic trafficking of mRNAs [98, 99]. YTHDC1 selectively associates with m^6^A, marks and modulates mRNA alternative splicing, and recruitments of YTHDF1 and YTHDF3 to m^6^A sites enhance mRNA translation [100]. In addition, some readers also participate in the modulation of mRNA stability. YTHDF2 targets RNA transcripts that contain m^6^A modifications for degradation [101], while the binding of IGF2BP1/2/3 to m^6^A-modified mRNA promotes mRNA stability and translation [102]. In summary, m^6^A modification tightly modulates most aspects of mRNA processing, including mRNA stability, pre-mRNA splicing, mRNA transportation, and translation.

As the most prevalent post-transcriptional modification, m^6^A is essential for pluripotency and reprogramming [20]. Transcriptome-wide m^6^A profiling has shown that the majority of key pluripotent genes (e.g. NANOG, Oct3/4, SOX2, and KLF4) have abundant m^6^A modifications on their RNA transcripts, which eventually impairs mRNA stability and induces RNA degradation [103, 104]. METTL3 is the core component of the m^6^A methyltransferase complex. Complete depletion of m^6^A in METTL3-null mice led to early embryonic lethality owing to prolonged RNA half-life of core pluripotency genes, resulting in a delay in initiation of differentiation programs [103]. Therefore, the correct deposition of m^6^A in RNA transcripts is essential for the maintenance of self-renewal capacity during embryo development.

Emerging evidence indicates that aberrant m^6^A profiles frequently occur in a variety of cancer types [105]. Unexpectedly, both elevated and depressed levels of m^6^A methylation have been reported in different types of cancer, such as liver cancer and acute myeloid leukemia (AML) [106]. In AML, elevated expression of METTL3 has been observed, which led to increased m^6^A methylation levels of BCL2 and c-MYC transcripts and thus enhanced their translation [107]. METTL3 also induced m^6^A modification within the mRNA transcript of SP1, an oncogene in AML which modulates c-MYC expression [108]. On the contrary, another group found that m^6^A demethylase FTO played an oncogenic role in AML through reducing the m^6^A levels of ABS2 and RARA, which led to decreased mRNA levels of these two targets and eventually contributed to leukemogenesis [109].

For CSCs, m^6^A demethylation actively helps to maintain the self-renewal capacity of cancer cells (Fig. 3b). In breast cancer cells, the m^6^A demethylase, ALKBH5, reduced the level of m^6^A modification in NANOG mRNA, which subsequently stabilized NANOG mRNA and thus promoted breast cancer stem cell phenotypes [33]. ALKBH5 was also highly expressed in glioblastoma stem-like cells (GSCs) and the knockdown of ALKBH5 attenuated the growth of patient-derived GSCs [35]. The mechanistic study revealed that ALKBH5 demethylated FOXM1 nascent RNA transcripts and enhanced FOXM1 expression, which ultimately maintained the self-renewal capacity of GSCs [35]. Similar to ALKBH5, another m^6^A demethylase FTO was reported to promote self-renewal and tumorigenesis in GSCs and suppression of FTO by its inhibitor MA2 attenuated GSC growth and self-renewal [34]. Consistently, treatment with another FTO inhibitor R-2HG significantly elevated global m^6^A modification in leukemia cells, which in turn induced the degradation of MYC/CEBPA RNA transcripts and inhibited the relevant pathways [55].

However, the opposite expression patterns of m^6^A exist in acute myeloid leukemia and glioblastoma. METTL14, a core component of the m^6^A methyltransferase complex, was dramatically elevated in normal hematopoietic stem/progenitor cells and acute myeloid leukemia cells [56]. METTL14 catalyzed the m^6^A modification in oncogenic factors MYC and MYB, increasing their mRNA stability and thus maintaining the stemness of leukemia stem cells [56]. In glioblastoma, m^6^A methyltransferase METTL3 was elevated in GSCs and its expression decreased during differentiation [54]. Subsequent studies found that SOX2 mRNA was methylated by METTL3 and that methylated SOX2 mRNA displayed prolonged stability, suggesting that HuR is essential for METTL3-mediated stabilization of SOX2 mRNA [54].

In summary, aberrant m^6^A modification frequently occurs in a variety of cancer types and m^6^A’s deregulation plays a vital role in modulating the stemness of CSCs. However, both elevated and depressed levels of m^6^A have been reported in CSCs, and the mechanisms by which m^6^A modification contributes to cell fate decisions remain elusive. Therefore, further studies are needed to explore the underlying molecular mechanisms.

## The underlying mechanisms for m^6^A in RNA expression and splicing

Currently, the mechanisms by which m^6^A methylation modulates mRNA decay are still under debate. The majority of the current findings indicate that m^6^A methylation predominantly hampers mRNA stability [110]. It was reported that m^6^A-modified mRNA has shorter half-live in mammalian cells [111]. Complete depletion of METTL3, the core component of the m^6^A methyltransferase complex, led to prolonged mRNA half-live when compared to that of wild type cells [111]. On the other hand, knockdown of m^6^A demethylase, ALKBH5, impaired the stability of NANOG and FOXM1 mRNA transcripts in CSCs [33, 35], indicating that m^6^A methylation might destabilize mRNA transcripts. Interestingly, recent studies suggest that the destabilizing effect of m^6^A is attributed to the cytosolic m^6^A reader protein YTHDF2. The carboxy-terminal of YTHDF2 preferentially binds to m^6^A-modified mRNAs, and its amino-terminal is responsible for the translocation of the m^6^A-modified mRNAs towards the P-body, where the unwanted mRNAs are degraded [17]. Furthermore, YTHDF2 silencing results in a prolonged lifetime of its mRNA targets, suggesting that YTHDF2 may play a vital role in mRNA decay [17].

In contrast to the mRNA-decay-promoting role of m^6^A methylation, a few emerging studies indicate that m^6^A methylation also stabilizes mRNA by recruiting IGF2BP1/2/3 and HuR proteins. For example, SOX2 is an m^6^A target for METTL3 and methylated SOX2 mRNA displays prolonged stability. In addition, RNA stabilizer protein HuR is essential for METTL3-mediated SOX2 mRNA stabilization [54]. On the contrary, IGF2BP1/2/3 proteins can recognize m^6^A-modified mRNAs and enhance the RNA stability of their target mRNAs in an m^6^A-dependent manner, thereby modulating cancer cell proliferation [102].

Therefore, it seems that the cellular fate of m^6^A-modified mRNA depends on their binding proteins. YTHDF2 recognizes the m^6^A-modified mRNA transcripts and initiates RNA degradation [101]. However, the binding of IGF2BP1/2/3 or HuR to m^6^A-modified mRNA enhances mRNA stability and translation [54, 102].

In addition to mRNA decay, the presence of m^6^A may also participate in mRNA alternative splicing. In 2016, two independent groups reported that m^6^A sites within the intron affected the splicing of Sxl gene [112, 113], a master regulator of Drosophila sex determination. The m^6^A mapping results revealed Sxl as a major intronic m^6^A target and that disruption of the m^6^A pathway compromised the female-specific Sxl splicing [112, 113]. Further studies demonstrated that m^6^A reader YT521-B was a dominant m^6^A effector for female-specific Sxl alternative splicing [112–114]. In mammalian cells, a few studies have found that m^6^A affected RNA splicing by recruiting m^6^A reader YTHDC1 to m^6^A-modified mRNA. Mechanistic studies showed that YTHDC1 modulated RNA alternative splicing through interacting with splicing factors [14, 115]. During mouse oocyte development, YTHDC1 regulates m^6^A-dependent processing of pre-mRNA transcripts through the recruitment of splicing factors CPSF6, SRSF3, and SRSF7. YTHDC1-deficient oocytes displayed extensive alternative polyadenylation, leading to altered 3′-UTR length [14]. In mammalian cells, genome-wide m^6^A mapping and PAR-CLIP showed that the binding sites of YTHDC1 and SRSF3 co-localized with m^6^A sites [115]. Subsequent studies found that YTHDC1 promoted exon inclusion by interacting with pre-mRNA splicing regulator SRSF3. To further investigate whether m^6^A modulates RNA splicing, various high-resolution m^6^A mapping methods have been used to determine whether m^6^A sites are located in the vicinity of splice junctions. Some groups have found enrichment of m^6^A in the proximity of exonic and intronic splice sites [116–118], while another independent group found that the majority of m^6^A sites were not located close to splice sites [111]. These contradicting results raise concerns over the accuracy of current approaches to m^6^A mapping. More studies will be needed to provide a precise mapping of m^6^A distribution within the nascent RNAs, which will eventually elucidate the role of m^6^A in RNA splicing.

As the most prevalent RNA modification form in eukaryotic mRNAs, the m^6^A-interacting proteins (writers, erasers, and readers) have been identified by serial biochemical approaches. Subsequent studies have highlighted the biological and pathological importance of these proteins. However, the underlying molecular mechanisms of m^6^A modifications need to be further explored. In conclusion, the central questions remain about how m^6^A is added on or removed from target mRNAs, and how m^6^A modulates RNA metabolism.

## Conclusion and perspectives

Previous studies highlighted mRNA modifications as key modulators in determining cell fate transition during embryonic development [103]. Recently, emerging evidence demonstrates that several mRNA modification forms are fundamental for maintaining the stemness of CSCs. A unique feature of CSCs is the efficient maintenance of their self-renewal capacity in response to external stimuli such as chemotherapy and radiotherapy. Therefore, depending on the distinct RNA modification profiles between CSCs and other tumor cells, we can exploit this unique feature to develop novel biomarkers to distinguish drug-resistant tumor cells from drug-responsive tumor cells. Furthermore, the dependency on RNA modifications to shift cancer cell fate may be able to be exploited as a powerful therapeutic strategy to specifically eliminate CSCs in cancer patients.

Recent breakthroughs in epitranscriptome sequencing technologies have enabled scientists to decode mRNA modifications in mammalian cells, which strengthen our current understanding of the distribution and function of various mRNA modifications. However, although more than 170 RNA modifications have been identified [105], only a few sequencing technologies have been established to decode RNA modifications. Moreover, many sequencing platforms fail to provide a precise transcriptome-wide RNA modification landscape at single-base resolution in eukaryotic cells. Thus, more robust and sensitive methods are urgently needed to decipher the epitranscriptome in mammalian cells. Recently, Nanopore technology, a novel single-molecule method, has displayed precise and single base-resolution detection of m^6^A in synthetic RNA molecules [13, 119]. This single-molecule approach might serve as a novel paradigm to detect different RNA modifications simultaneously.

In addition to novel sequencing strategies, the corresponding RNA modifying enzymes remain largely unknown. For instance, although the m^5^C methyltransferase NSUN2 has been characterized, we still do not know the parallel demethylases which are responsible for the removal of m^5^C [77]. Moreover, although the aberrant expression of RNA modifying enzyme has been identified in most aspects of cancer cells, it remains largely unknown how specific RNA modifications affect distinct cancer cell sub-populations. The functional consequences of RNA modification disruption remain unclear. Thus, a detailed understanding of how RNA modifications influence cancer cell fate is essential for harnessing these findings into novel cancer therapies.

In conclusion, the aberrant deposition of RNA modifications is tightly linked to the stemness of CSCs. The underlying molecular mechanisms show that RNA modifications orchestrate almost every step of mRNA metabolism, ranging from mRNA biogenesis to mRNA decay, which can eventually converge to determine the cancer stem cell’s fate and tumor progression.

## Data Availability

Not applicable.

## Abbreviations

ALKBH5: Alkb homologue 5
CRC: Colorectal cancer
CSCs: Cancer stem cells
D2-HG: D2-hydorxyglutarate
DNMT 1: DNA methyltransferase 1
DNMT2: DNA methyltransferase 2
FOXM1: Forkhead box protein M1
FOXM1-AS: Antisense to FOXM1
FTO: Fat mass and obesity associated protein
GSCs: Glioblastoma stem-like cells
HCC: Hepatocellular carcinoma
HuR: Human antigen R
IGF2BP: IGF2 mRNA binding proteins
lncRNAs: Long non-coding RNAs
LSCs: Leukemia stem cells
m^5^C: 5-methylcytosine
m^6^A: N 6-methyladenosine
METTL3: Methyltransferase-like 3
miRNAs: Micro RNAs
mRNAs: Messenger RNAs
ncRNAs: Noncoding RNAs
PAR-CLIP: Photoactivatable-Ribonucleoside-Enhanced Crosslinking and Immunoprecipitation
R-2HG: R-2-hydroxyglutarate
Sxl: Sex lethal
UTR: Untranslated terminal region
WTAP: Wilms tumor 1-associated protein
YBX1: Y-box binding protein 1
